# Life truncated multiple dependent state plan for imprecise Weibull distributed data

**DOI:** 10.1038/s41598-024-55694-2

**Published:** 2024-03-26

**Authors:** Gadde Srinivasa Rao, Muhammad Aslam, Peter Kirigiti Josephat, Zainalabideen Al-Husseini, Mohammed Albassam

**Affiliations:** 1 Department of Mathematics and Statistics, University of Dodoma, PO. Box: 259, Dodoma, Tanzania; 2 Department of Statistics, Faculty of Science, King Abdulaziz University, 21551 Jeddah, Saudi Arabia; 3 Department of Accounting, College of Adminstrative Sciences, Al-Mustaqbal University, Babylon, 51001 Iraq

**Keywords:** Single sampling plan, Multiple dependent state, Classical statistics, Indeterminacy, Luminous intensities of diodes, Engineering, Mathematics and computing

## Abstract

This paper aims to provide a multiple dependent state (MDS) sampling technique for light-emitting diode luminous intensities under indeterminacy by employing time truncated sampling schemes and the Weibull distribution. This indicates that ASN is significantly impacted by the indeterminacy parameter. Furthermore, a comparison is shown between the existing, indeterminate sampling plans and the recommended sample designs. The projected sampling technique is illustrated by calculating the luminous intensities of LEDs using the Weibull distribution. Based on the findings and practical example, we conclude that the suggested strategy needs a smaller sample size than SSP and the current MDS sampling plan.

## Introduction

The luminous brightness of light-emitting diodes changes randomly and conforms to a statistical distribution. The Weibull distribution is one of the statistical distributions used extensively for dependability, engineering application research, and estimates. When the parameters or observations are known, traditional statistics are used for estimation and prediction. Light-emitting diode data is often reported in terms of their luminous intensities over time. Using the present distributions is not possible in this situation.

Many authors have created a time-condensed life test based on the conventional acceptance sampling plan using various life distributions. A few sources on acceptance sampling strategies include^1–9^. Recently, several scholars have focused on a range of sampling plans including single sampling plans (SSP) and multiple dependent state (MDS) sampling plans for various distributions. The MDS sampling plan's process was started by^10^ and according to his explanation, "the MDS sampling plan is known as an attribute inspection procedure where the decision is made for each lot based on one of the three conditions namely accept the lot; reject the lot; or conditionally accept or reject the lot based on the disposition of future related lots." Later, a large number of authors investigated MDS sampling designs for a variety of distributions, including^11–26^.

The previously described sample approaches do not provide background information on the measure of indeterminacy because they blend classical statistics with a fuzzy environment. The measurements of determinacy, indeterminacy, and falseness are described in depth in the neutrosophic logic, see^27^. The concepts of neutrosophic statistics^28–30^ were introduced using the idea of neutrosophic logic. For this reason, fuzzy logic and interval-based analysis are less successful than neutrosophic logic. On the basis of a fuzzy environment^31^,^32^ created a single sampling plan. The results of sampling error on evaluation based on a fuzzy environment were reported by^32^. Please see^33–36^ and for more authors who explored the single plan employing a fuzzy logic environment^36^. Information on the determinacy and indeterminacy measures can be found in the neutrosophic statistics, see^37^. In case the indeterminacy measure is not documented, classical statistics takes over in neutrosophic statistics. By using neutrosophic statistics^38–40^, offered acceptance sample strategies. Aslam et al.^41^ worked on group plan for Weibull distribution. Neutrosophic Weibull and the neutrosophic family of Weibull distribution were studied by^42^. Woodall et al.^43^ suggested to use determinate sample size in designing sampling plans under neutrosophic statistics.

The existing sample plans, which rely on conventional statistics and fuzzy logic, do not offer information on the measure of indeterminacy. Upon reviewing the current research literature on sampling plans, we believe it is groundbreaking that no one has studied the MDS sample plan for the Weibull distribution under indeterminacy. The present work aims at testing light-emitting diode luminous intensities under indeterminacy by employing an MDS sampling approach for the Weibull distribution. It is expected that the proposed sampling design demonstrates a smaller ASN than the current sampling designs, hence testing the luminous intensities of light-emitting diodes.

We demonstrated the MDS sampling plan for the Weibull distribution under indeterminacy in Section “Methodologies”. Section “Comparative studies” presented a comparison of existing indeterminacy sampling strategies and existing classical sampling plans. A real-world scenario with the luminous intensities of light-emitting diodes is used in Section “LED manufacturing process data illustration” to illustrate the proposed sampling plan for the indeterminacy. The conclusions and upcoming research projects are covered in Section “Concluding remarks”.

## Methodologies

Aslam^44^ introduced neutrosophic Weibull distribution that will be recalled in this section. We will also provide the architecture of the sample plan for determining the mean luminosities of light-emitting diodes in unclear conditions.

Consider the neutrosophic probability density function (NPDF) $f\left({x}_{N}\right)=f\left({x}_{L}\right)+f\left({x}_{U}\right){I}_{N};{I}_{N}\epsilon \left[{I}_{L},{I}_{U}\right]$ which has a determinate part $f\left({x}_{L}\right)$, an indeterminate part $f\left({x}_{U}\right){I}_{N}$ and indeterminacy interval ${I}_{N}\epsilon \left[{I}_{L},{I}_{U}\right]$. It should be noted that the neutrosophic random variable ${x}_{N}\epsilon \left[{x}_{L},{x}_{U}\right]$ follows the NPDF. The generalization of the PDF under classical statistics is the NPDF. When ${I}_{L}$=0 the classical statistics of the proposed neutrosophic form of $f\left({x}_{N}\right)\epsilon \left[f\left({x}_{L}\right),f\left({x}_{U}\right)\right]$ simplifies to PDF. The Weibull distribution's NPDF is defined as follows using this information.

1 $$f\left({x}_{N}\right)=\left\{\left(\frac{\beta }{\alpha }\right){\left(\frac{{x}_{N}}{\alpha }\right)}^{\beta -1}{e}^{-{\left(\frac{{x}_{N}}{\alpha }\right)}^{\beta }}\right\}+\left\{\left(\frac{\beta }{\alpha }\right){\left(\frac{{x}_{N}}{\alpha }\right)}^{\beta -1}{e}^{-{\left(\frac{{x}_{N}}{\alpha }\right)}^{\beta }}\right\}{I}_{N}; {I}_{N}\epsilon \left[{I}_{L},{I}_{U}\right]$$

where $\alpha$ and $\beta$ are scale and shape parameters, accordingly. Here, it should be noted that the Weibull distribution's proposed NPDF is a generalization of its PDF in terms of classical statistics. When ${I}_{L}$=0 the neutrosophic Weibull distribution's NPDF simplifies to the Weibull distribution. The Weibull distribution's neutrosophic cumulative distribution function (NCDF) is given by

2 $$F\left({x}_{N}\right)=1-\left\{{e}^{-{\left(\frac{{x}_{N}}{\alpha }\right)}^{\beta }}\left(1+{I}_{N}\right)\right\}+{I}_{N}; {I}_{N}\epsilon \left[{I}_{L},{I}_{U}\right]$$

The Weibull distribution's neutrosophic mean is given by

3 $${\mu }_{N}=\alpha\Gamma \left(1+1/\beta \right)\left(1+{I}_{N}\right); {I}_{N}\epsilon \left[{I}_{L},{I}_{U}\right]$$

The neutrosophic Weibull distribution's median life is given by

4 $${\widetilde{\mu }}_{N}=\alpha {\left({\text{ln}}(2)\right)}^{1/\beta }\left(1+{I}_{N}\right); {I}_{N}\epsilon \left[{I}_{L},{I}_{U}\right]$$

Balamurali et al.^22^provide the following well-designed methodology for MDS sampling design, and under neutrosophic statistics proposed by^45^.

The following are the alternative and null hypotheses for the average luminous intensities of light-emitting diodes:

$${H}_{0}:\mu ={\mu }_{0}$$ Vs. $${H}_{1}:\mu \ne {\mu }_{0}$$

Where ${\mu }_{0}$ denotes the desired average wind speed and $\mu$ represents the actual average wind speed. According to these data, the suggested sample approach is presented as follows:

*Step 1*: Pick a sample from the batch that is size *n*. These samples were put through a life test for a set amount of time ${t}_{N0}$. Mention the average ${\mu }_{0N}$ and the amount of indeterminacy ${I}_{N}\epsilon \left[{I}_{L},{I}_{U}\right]$.

*Step 2*: The test ${H}_{0}:{\mu }_{N}={\mu }_{0N}$ could be accepted if the average daily number of cases for ${c}_{1}$ days are greater or equal to ${\mu }_{0}$ (i.e.,${\mu }_{0N}\le {c}_{1}$). If average daily number of cases in ${c}_{2}$ days are less than to ${\mu }_{0}$ (i.e., ${\mu }_{0}$>${c}_{2}$) then test ${H}_{0}:{\mu }_{N}={\mu }_{0N}$ could be rejected and come to an end the test, where ${c}_{1}\le {c}_{2}$.

*Step 3*: When ${{c}_{1}<\mu }_{0N}\le {c}_{2}$ then accept the current lot if *m* preceding lots, the mean number of cases must be less than or equal to ${c}_{1}$ before the test termination time ${t}_{N0}$.

The proposed plan has four values, namely, $n, \, {c}_{1}, \, {c}_{2}$ and *m* where *n* is the sample size, and $c_{1}$ is the maximum number of allowable items that failed for unconditional acceptance ${c}_{1}$, ${c}_{2}$ is the maximum number of additional items that failed for conditional acceptance ${c}_{1}\le {c}_{2}$, and *m* is the number of subsequent lots (prior) required to reach a conclusion. The characteristics of the MDS sampling plan converge to $m \to \infty$ and/or ${c}_{1}={c}_{2}=c$ (say), and MDS oversimplifies SSP. The OC function can be used to determine the concert of any sampling design.

Using the binomial chance law, the OC function for an MDS sample design based on WD is expressed as follows:

5 $$P_{a} (p) = \sum\limits_{d = 0}^{{c_{1} }} {\left( \begin{gathered} n \hfill \\ d \hfill \\ \end{gathered} \right)} \,p^{d} \,(1 - p)^{(n - d)} + \sum\limits_{{d = c_{1} + 1}}^{{c_{2} }} {\left( \begin{gathered} n \hfill \\ d \hfill \\ \end{gathered} \right)} \,p^{d} \,(1 - p)^{(n - d)} \times \left[ {\sum\limits_{d = 0}^{{c_{1} }} {\left( \begin{gathered} n \hfill \\ d \hfill \\ \end{gathered} \right)} \,p^{d} \,(1 - p)^{(n - d)} } \right]^{m}$$

Suppose that ${t}_{0}=a{\mu }_{N0}$ be the time in days, where $a$ is the termination ratio. The probability of accepting ${H}_{0}:{\mu }_{N}={\mu }_{N0}$ is given by

6 $$P\left({p}_{N}\right)=\sum_{i=0}^{c}\left(\begin{array}{c}n\\ i\end{array}\right){p}_{N}^{i}{\left(1-{p}_{N}\right)}^{n-i}$$

where ${p}_{N}$ is the probability of rejecting ${H}_{0}:{\mu }_{N}={\mu }_{N0}$ and obtained using Eqs. (2) and (3) as ${p}_{N}=F\left({t}_{N}\le {t}_{N0}\right)$ and defined by

7 $${p}_{N}=1-\left\{{\text{exp}}(-{a}^{\beta }{\left({\mu }_{N}/{\mu }_{N0}\right)}^{-\beta }{{\left(\Gamma \left(1/\beta \right)/\beta \right)}^{\beta }\left(1+{I}_{N}\right)}^{\beta })\left(1+{I}_{N}\right)\right\}+{I}_{N}$$

where ${\mu }_{N}/{\mu }_{N0}$ is the difference between the specified average luminous intensities of light-emitting diodes and the actual average luminous intensities of light-emitting diodes. Assume that $\gamma$ and $\delta$ be type-I and type-II errors, respectively. The proposed plan for testing ${H}_{0}:{\mu }_{N}={\mu }_{N0}$ N0 is one that the meteorologists are interested in using because it ensures that the probability of accepting ${H}_{0}:{\mu }_{N}={\mu }_{N0}$ when it is true should be greater than $1-\gamma$ at ${\mu }_{N}/{\mu }_{N0}$ and the probability of accepting ${H}_{0}:{\mu }_{N}={\mu }_{N0}$ when it is incorrect should be lower than $\delta$ at ${\mu }_{N}/{\mu }_{N0}=1$. The following two inequalities will be satisfied by the plan parameters for testing ${H}_{0}:{\mu }_{N}={\mu }_{N0}$.

8 $${P}_{a}\left({p}_{1N}|{\mu }_{N}/{\mu }_{N0}\right)\ge 1-\gamma$$

9 $${P}_{a}\left({p}_{2N}|{\mu }_{N}/{\mu }_{N0}=1\right)\le \delta$$

where ${p}_{1N}$ and ${p}_{2N}$ are defined by

10 $${p}_{1N}=1-\left\{{\text{exp}}(-{a}^{\beta }{\left({\mu }_{N}/{\mu }_{N0}\right)}^{-\beta }{{\left(\Gamma \left(1/\beta \right)/\beta \right)}^{\beta }\left(1+{I}_{N}\right)}^{\beta })\left(1+{I}_{N}\right)\right\}+{I}_{N}$$

and

11 $${p}_{2N}=1-\left\{{\text{exp}}(-{a}^{\beta }{{\left(\Gamma \left(1/\beta \right)/\beta \right)}^{\beta }\left(1+{I}_{N}\right)}^{\beta })\left(1+{I}_{N}\right)\right\}+{I}_{N}$$

The average sample size (ASN) has often been decreased by the use of on-hand sampling techniques. Any sample strategy's main objective is usually to lower the ASN, which also helps to lower the amount of time and money needed for the inspection. Accordingly, the goal of the suggested MDS sample design is to lower the ASN for WD in the suggested scenario. The non-linear programming approach yields the optimal quantities, which are stated as follows:

Minimize $$ASN(p_{1N} ) = n$$

Subject to $$P_{a} (p_{1N} ) \ge 1 - \gamma$$

$$P_{a} (p_{2N} ) \le \delta$$

12 $$n > 1,\,m \ge 1,\,c_{2} > c_{1} \ge 0.$$

where$p_{1N}$ and $p_{2N}$ are the likelihood of failure at producer’s and consumer’s risks respectively. These acceptance probabilities might be calculated by using the following equations:

13 $$P_{a} (p_{1N} ) = \sum\limits_{d = 0}^{{c_{1} }} {\left( \begin{gathered} n \hfill \\ d \hfill \\ \end{gathered} \right)} \,p_{1N}^{d} \,(1 - p_{1N} )^{(n - d)} + \sum\limits_{{d = c_{1} + 1}}^{{c_{2} }} {\left( \begin{gathered} n \hfill \\ d \hfill \\ \end{gathered} \right)} \,p_{1N}^{d} \,(1 - p_{1N} )^{(n - d)} \left[ {\sum\limits_{d = 0}^{{c_{1} }} {\left( \begin{gathered} n \hfill \\ d \hfill \\ \end{gathered} \right)} \,p_{1N}^{d} (1 - p_{1N} )^{(n - d)} } \right]^{m}$$

and

14 $$P_{a} (p_{2N} ) = \sum\limits_{d = 0}^{{c_{1} }} {\left( \begin{gathered} n \hfill \\ d \hfill \\ \end{gathered} \right)} \,p_{2N}^{d} \,(1 - p_{2N} )^{(n - d)} + \sum\limits_{{d = c_{1} + 1}}^{{c_{2} }} {\left( \begin{gathered} n \hfill \\ d \hfill \\ \end{gathered} \right)} \,p_{2N}^{d} \,(1 - p_{2N} )^{(n - d)} \left[ {\sum\limits_{d = 0}^{{c_{1} }} {\left( \begin{gathered} n \hfill \\ d \hfill \\ \end{gathered} \right)} \,p_{2N}^{d} (1 - p_{2N} )^{(n - d)} } \right]^{m}$$

The proposed plan consists of parameters $c_{1} ,c_{2} ,m\,\,{\text{and}}\,{\text{ASN}}$ that are obtained by solving the non-linear programming problem in Eq. (12) for $\delta =\left\{\mathrm{0.25,0.10,0.05}\right\}$, $\gamma =0.10$, $a=0.5, 1.0$ and known ${I}_{N}$ are placed in Tables 1, 2, 3, and 4. Tables 1 and 2 show the WD for $\beta =2$, while Tables 3 and 4 show the WD with $\beta =1$(exponential distribution). The following points can be drawn from the results in the tables.

- As the value of $a$ increases from 0.5 to 1.0, the value of ASN decreases
- When all other parameters are held constant, ASN decreases as the shape parameter increases from $\beta$ = 1 to $\beta$ = 2.
- Furthermore, it is discovered that the indeterminacy parameter ${I}_{N}$ has a significant influence on minimizing ASN values.

**Table 1:** The MDS design values for $\beta =2$ and $a=0.5$.

$\delta$	$\frac{{\upmu }_{{\text{N}}}}{{\upmu }_{0{\text{N}}}}$	${I}_{L}$=0.00					${I}_{L}$=0.02					${I}_{L}$=0.04					${I}_{L}$=0.05				
		${c}_{1}$	${c}_{2}$	$m$	$L\left({p}_{1}\right)$	ASN	${c}_{1}$	${c}_{2}$	$m$	$L\left({p}_{1}\right)$	ASN	${c}_{1}$	${c}_{2}$	$m$	$L\left({p}_{1}\right)$	ASN	${c}_{1}$	${c}_{2}$	$m$	$L\left({p}_{1}\right)$	ASN
0.25	1.5	3	13	3	0.9001	28	3	5	2	0.9015	27	3	13	3	0.9005	25	3	7	2	0.9155	25
	1.6	2	4	2	0.9024	22	2	8	2	0.9111	21	2	12	2	0.9086	20	2	4	2	0.9048	19
	1.7	2	4	2	0.9369	22	2	3	3	0.9080	20	2	6	3	0.9342	19	2	4	2	0.9387	19
	1.8	1	3	2	0.9083	15	1	3	1	0.9198	14	1	11	2	0.9025	14	1	3	2	0.9087	13
	1.9	1	3	2	0.9322	15	1	2	2	0.9118	14	1	11	3	0.9203	13	1	3	2	0.9326	13
	2.0	1	3	2	0.9497	15	1	2	2	0.9324	14	1	11	3	0.9404	13	1	3	2	0.9500	13
0.1	1.5	5	10	1	0.9095	54	5	8	1	0.9001	50	5	8	1	0.9035	47	5	8	1	0.9003	46
	1.6	4	14	2	0.9119	44	4	13	2	0.9177	41	4	14	2	0.9151	39	4	14	2	0.9144	38
	1.7	3	6	2	0.9165	36	3	7	2	0.9203	34	3	6	2	0.9182	32	3	5	2	0.9075	31
	1.8	2	4	1	0.9119	30	2	6	2	0.9016	27	2	4	1	0.9092	27	2	4	1	0.9125	26
	1.9	2	12	2	0.9302	29	2	6	2	0.9334	27	2	3	1	0.9080	26	2	12	2	0.9319	25
	2.0	2	12	2	0.9528	29	1	3	1	0.9022	21	1	3	1	0.9000	20	1	4	1	0.9046	20
0.05	1.5	7	10	1	0.9010	73	7	11	1	0.9170	69	7	10	1	0.9033	65	7	10	1	0.9056	63
	1.6	5	8	1	0.9155	58	5	8	1	0.9137	55	5	8	1	0.9138	52	5	15	2	0.9017	49
	1.7	4	11	2	0.9154	49	4	6	1	0.9152	47	4	14	2	0.9127	44	4	14	2	0.9106	43
	1.8	3	13	2	0.9045	42	3	13	2	0.9111	39	3	13	2	0.9096	37	3	13	2	0.9094	36
	1.9	2	6	1	0.9100	36	2	6	1	0.9097	34	2	6	1	0.9109	32	2	5	1	0.9065	31
	2.0	2	12	2	0.9127	34	2	12	2	0.9135	32	2	12	2	0.9159	30	2	12	2	0.9177	29

**Table 2:** The MDS design values for $\beta =2$ and $a=1.0$.

$\delta$	$\frac{{\upmu }_{{\text{N}}}}{{\upmu }_{0{\text{N}}}}$	${I}_{L}$=0.00					${I}_{L}$=0.02					${I}_{L}$=0.04					${I}_{L}$=0.05				
		${c}_{1}$	${c}_{2}$	$m$	$L\left({p}_{1}\right)$	ASN	${c}_{1}$	${c}_{2}$	$m$	$L\left({p}_{1}\right)$	ASN	${c}_{1}$	${c}_{2}$	$m$	$L\left({p}_{1}\right)$	ASN	${c}_{1}$	${c}_{2}$	$m$	$L\left({p}_{1}\right)$	ASN
0.25	1.5	4	5	1	0.9026	11	3	7	1	0.9126	9	4	5	1	0.9036	10	3	5	1	0.9159	8
	1.6	2	4	1	0.9132	7	2	12	1	0.9068	7	3	4	1	0.9231	8	3	5	1	0.9521	8
	1.7	2	4	1	0.9440	7	2	3	2	0.9200	6	2	3	1	0.9254	6	2	3	1	0.9178	6
	1.8	1	4	1	0.9131	5	1	3	2	0.9092	4	1	2	1	0.9059	4	2	3	1	0.9434	6
	1.9	1	4	1	0.9355	5	1	3	2	0.9328	4	1	2	1	0.9288	4	1	2	1	0.9230	4
	2.0	1	4	1	0.9519	5	1	3	2	0.9501	4	1	2	1	0.9459	4	1	2	1	0.9413	4
0.1	1.5	6	16	2	0.9210	17	5	9	1	0.9004	15	5	8	1	0.9038	14	6	16	2	0.9224	15
	1.6	4	6	1	0.9163	13	4	14	2	0.9121	12	4	7	1	0.9264	12	4	14	2	0.9188	11
	1.7	3	5	1	0.9151	11	3	13	2	0.9087	10	3	6	1	0.9288	10	3	13	2	0.9219	9
	1.8	2	6	1	0.9079	9	3	4	1	0.9248	10	2	6	1	0.9120	8	2	12	2	0.9019	7
	1.9	2	4	2	0.9264	8	2	3	1	0.9036	8	2	6	1	0.9403	8	2	12	2	0.9337	7
	2.0	2	4	2	0.9499	8	2	3	1	0.9302	8	2	6	1	0.9596	8	2	12	2	0.9553	7
0.05	1.5	7	13	1	0.9041	22	7	17	1	0.9011	21	8	10	1	0.9132	21	7	12	1	0.9168	19
	1.6	5	8	1	0.9089	17	5	8	1	0.9144	16	6	8	1	0.9290	17	5	9	1	0.9176	15
	1.7	4	14	2	0.9109	14	4	8	1	0.9297	14	4	7	1	0.9338	13	4	9	1	0.9301	13
	1.8	3	5	1	0.9181	12	3	13	2	0.9090	11	3	6	1	0.9284	11	3	5	2	0.9059	10
	1.9	2	5	1	0.9008	10	2	4	1	0.9058	9	2	6	1	0.9032	9	3	5	2	0.9412	10
	2.0	2	5	1	0.9310	10	2	4	1	0.9346	9	2	6	1	0.9326	9	2	12	2	0.9191	8

**Table 3:** The MDS design values for $\beta =1$ and $a=0.50$.

$\delta$	$\frac{{\upmu }_{{\text{N}}}}{{\upmu }_{0{\text{N}}}}$	${I}_{L}$=0.00					${I}_{L}$=0.02					${I}_{L}$=0.04					${I}_{L}$=0.05				
		${c}_{1}$	${c}_{2}$	$m$	$L\left({p}_{1}\right)$	ASN	${c}_{1}$	${c}_{2}$	$m$	$L\left({p}_{1}\right)$	ASN	${c}_{1}$	${c}_{2}$	$m$	$L\left({p}_{1}\right)$	ASN	${c}_{1}$	${c}_{2}$	$m$	$L\left({p}_{1}\right)$	ASN
0.25	1.5	14	18	1	0.9002	45	14	24	2	0.9065	42	13	17	1	0.9003	39	13	19	2	0.9028	37
	1.6	11	17	2	0.9215	35	10	15	2	0.9090	31	10	17	2	0.9098	30	10	14	1	0.9047	31
	1.7	8	18	2	0.9050	27	8	18	2	0.9065	26	8	18	2	0.9098	25	8	18	3	0.9001	24
	1.8	6	10	2	0.9030	21	6	11	1	0.9036	22	6	10	1	0.9059	21	6	16	1	0.9015	21
	1.9	5	10	1	0.9032	20	5	9	1	0.9081	19	5	15	2	0.9051	17	5	9	1	0.9089	18
	2.0	5	7	2	0.9155	18	5	7	3	0.9087	17	5	15	2	0.9284	17	4	14	2	0.9013	14
0.1	1.5	24	29	1	0.9018	78	24	31	1	0.9074	76	23	32	1	0.9013	71	23	29	1	0.9031	69
	1.6	17	24	1	0.9012	59	17	25	1	0.9023	57	17	25	1	0.9028	55	17	25	1	0.9040	54
	1.7	14	20	1	0.9170	50	14	19	1	0.9150	48	13	20	1	0.9039	44	13	19	1	0.9061	43
	1.8	11	16	1	0.9144	41	11	20	2	0.9062	38	11	16	1	0.9183	38	11	21	2	0.9077	36
	1.9	9	13	1	0.9083	35	9	14	1	0.9116	34	9	13	2	0.9034	31	9	14	1	0.9173	32
	2.0	8	18	2	0.9045	31	8	18	2	0.9019	30	8	18	2	0.9007	29	8	18	2	0.9137	28
0.05	1.5	31	40	1	0.9024	103	30	38	1	0.9007	96	31	38	1	0.9077	95	30	38	1	0.9027	91
	1.6	23	31	1	0.9053	80	23	28	1	0.9028	76	23	31	1	0.9136	74	22	29	1	0.9017	70
	1.7	18	25	1	0.9135	65	18	23	1	0.9116	62	17	22	1	0.9029	57	17	22	1	0.9034	56
	1.8	14	20	1	0.9079	53	14	20	1	0.9101	51	14	19	1	0.9076	49	14	19	1	0.9107	48
	1.9	12	17	1	0.9114	47	12	17	1	0.9175	45	12	16	1	0.9154	43	12	15	1	0.9023	42
	2.0	10	15	1	0.9100	41	10	14	1	0.9110	39	10	16	1	0.9176	38	10	15	1	0.9200	37

**Table 4:** The MDS design values for $\beta =1$ and $a=1.0$.

$\delta$	$\frac{{\upmu }_{{\text{N}}}}{{\upmu }_{0{\text{N}}}}$	${I}_{L}$=0.00					${I}_{L}$=0.02					${I}_{L}$=0.04					${I}_{L}$=0.05				
		${c}_{1}$	${c}_{2}$	$m$	$L\left({p}_{1}\right)$	ASN	${c}_{1}$	${c}_{2}$	$m$	$L\left({p}_{1}\right)$	ASN	${c}_{1}$	${c}_{2}$	$m$	$L\left({p}_{1}\right)$	ASN	${c}_{1}$	${c}_{2}$	$m$	$L\left({p}_{1}\right)$	ASN
0.25	1.5	14	24	2	0.9031	26	14	18	1	0.9048	26	15	25	2	0.9143	26	14	24	2	0.9063	24
	1.6	11	21	2	0.9117	21	11	13	2	0.9024	20	10	20	2	0.9044	18	11	13	2	0.9103	19
	1.7	9	11	1	0.9060	18	8	11	1	0.9065	16	9	19	4	0.9082	16	9	12	2	0.9299	16
	1.8	7	9	2	0.9078	14	7	9	1	0.9156	14	7	9	2	0.9188	13	6	9	1	0.9062	12
	1.9	6	8	1	0.9092	13	5	8	1	0.9027	11	5	15	2	0.9006	10	6	9	1	0.9315	12
	2.0	5	15	2	0.9046	11	5	15	4	0.9023	10	4	8	1	0.9014	9	5	15	2	0.9150	10
0.1	1.5	25	29	1	0.9006	48	24	29	1	0.9015	45	23	29	1	0.9010	42	24	31	1	0.9106	43
	1.6	19	25	2	0.9095	37	18	23	2	0.9024	34	17	21	1	0.9012	32	18	22	1	0.9159	33
	1.7	15	18	2	0.9006	30	15	20	2	0.9182	29	14	17	1	0.9046	27	13	17	1	0.9073	25
	1.8	11	15	1	0.9006	24	11	15	1	0.9107	23	11	14	1	0.9090	22	11	15	2	0.9098	21
	1.9	10	13	1	0.9147	22	10	12	1	0.9000	21	9	13	1	0.9086	19	9	19	1	0.9004	19
	2.0	8	18	1	0.9031	19	8	12	1	0.9137	18	8	11	1	0.9223	17	8	12	1	0.9207	17
0.05	1.5	34	41	1	0.9152	66	32	37	1	0.9027	60	31	40	1	0.9014	57	30	36	1	0.9014	54
	1.6	24	32	1	0.9023	49	23	28	1	0.9023	45	23	32	1	0.9024	44	22	27	1	0.9048	41
	1.7	19	26	1	0.9129	40	15	18	1	0.9049	31	18	22	1	0.9118	35	17	22	1	0.9044	33
	1.8	15	22	1	0.9060	33	12	16	1	0.9127	26	15	18	1	0.9067	30	14	18	1	0.9066	28
	1.9	12	16	1	0.9077	27	10	17	1	0.9018	23	12	15	1	0.9042	25	11	15	1	0.9009	23
	2.0	11	14	1	0.9173	25	9	13	1	0.9142	23	10	16	1	0.9115	22	11	15	1	0.9337	23

## Comparative studies

This section discusses the suggested plan's effectiveness in terms of ASN. The lower the sample size, the less expensive it is to test the luminous intensity hypothesis for average LEDs. The suggested sample plan is the expansion of the plan under classical statistics if there is no uncertainty or indeterminacy in the recording of the average LED's luminous intensity. When ${I}_{N}$=0, the suggested sampling plan lowers to the current sampling plan. The plan parameters under the classical statistics are shown in the first column of Tables 1, 2, 3, and 4.

**Figure 1:**
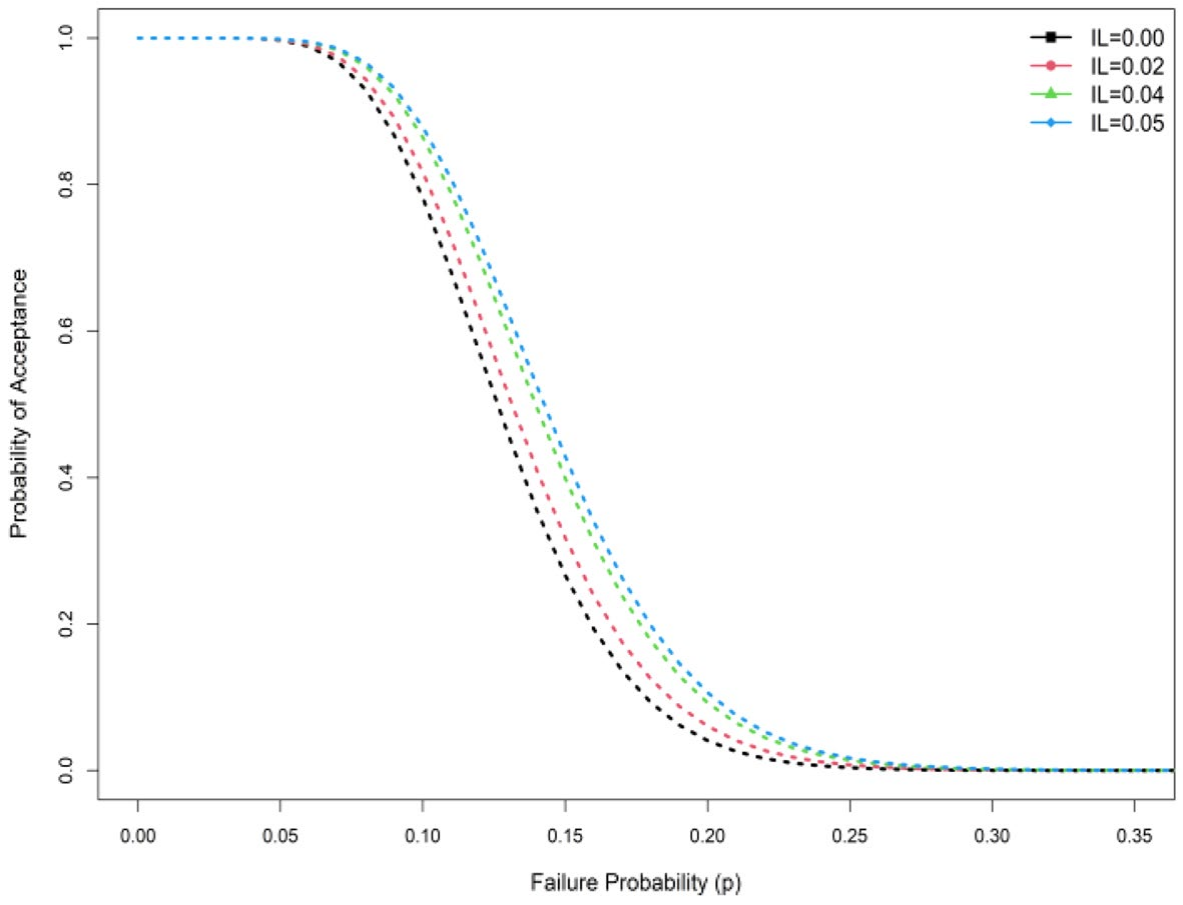
At various indeterminacy values, the OC curve plan.

**Figure 2:**
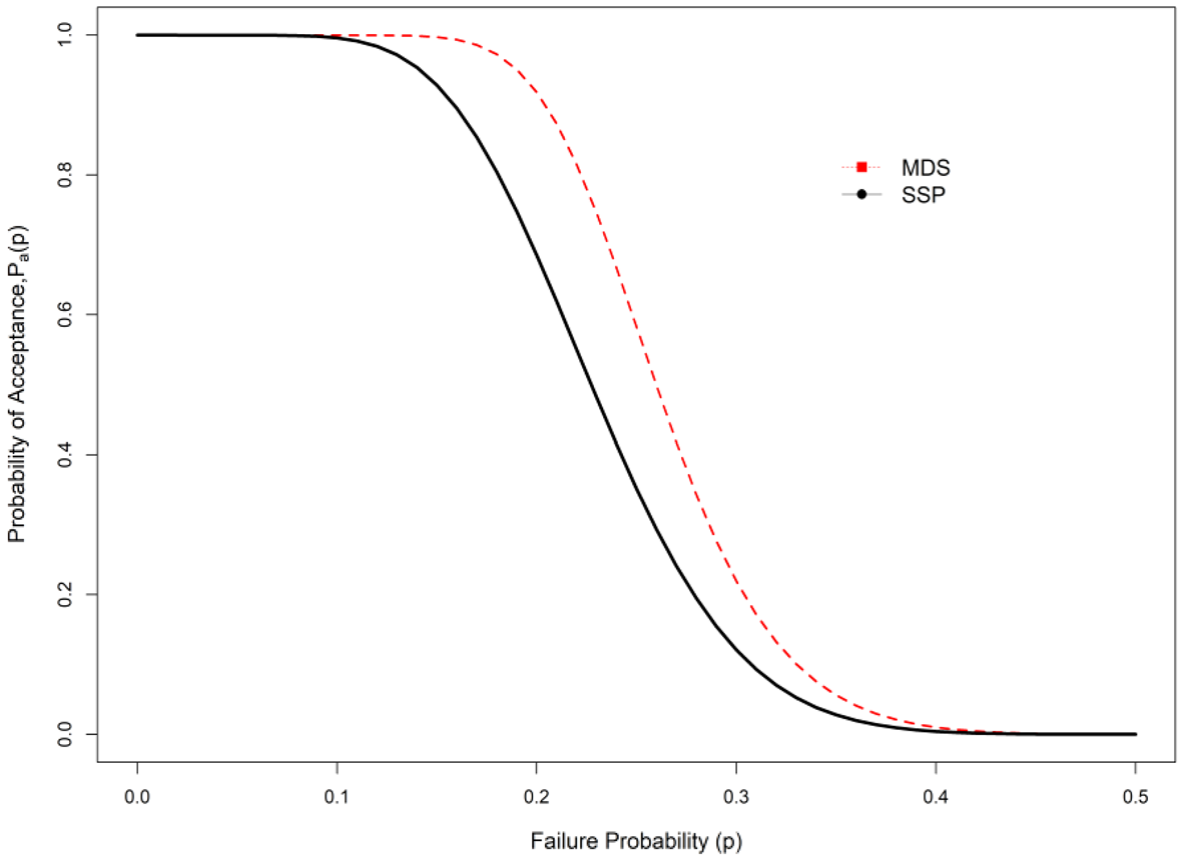
At various indeterminacy values, the OC curve of SSP and MDS.

**Figure 3:**
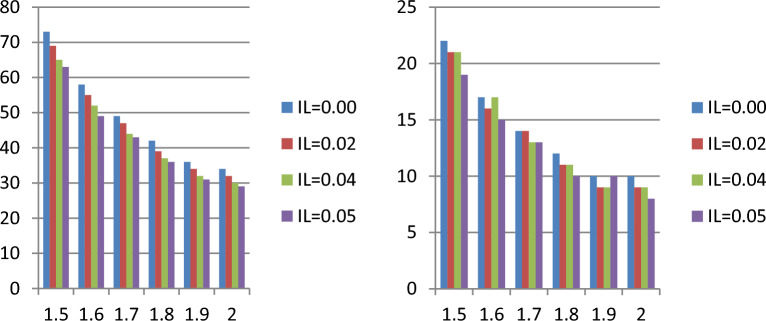
The ASN performance of MDS design values for $\beta =2$ and $\delta$ =0.05. (Left side $a=0.5$ and right side $a=1.0$).

**Figure 4:**
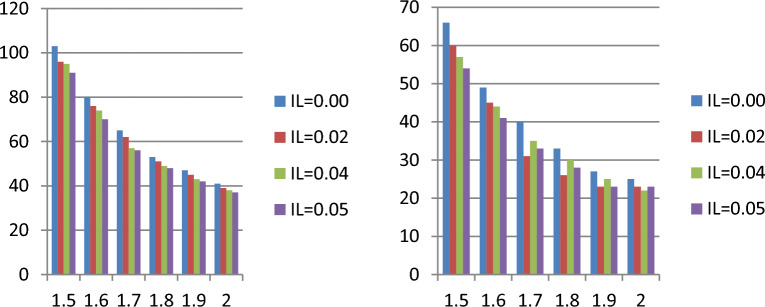
The ASN performance of MDS design values for $\beta =1$ and $\delta$ =0.05. (Left side $a=0.5$ and right side $a=1.0$).

Tables 1, 2, 3, and 4 show that when the indeterminacy parameter ${I}_{N}$ rises, lower values of the ASN are needed to test ${H}_{0}:{\mu }_{N}={\mu }_{N0}$ . For instance, it can be observed that ASN = 54 from the plan under classical statistics and ASN = 46 for the suggested sample plan when ${I}_{L}$=0.05 from Table 1 when ${\mu }_{N}/{\mu }_{N0}$=1.5, $\delta$ =0.10, $\gamma =0.10$, *a* = 0.5 and $\beta$ =2. According to the study, the existing sample plan under classical statistics is less effective in ASN than the proposed plan under indeterminacy. Additionally, as the WD transforms into an exponential distribution when $\beta$ =1 for comparison purposes, we created Tables 3 and 4. Tables 1, 2, 3, and 4 illustrate that the WD shows fewer samples than the exponential distribution. For instance, Table 3 shows that the ASN is 39 when the recommended plan values are ASN = 25 for $\beta$ =2 and when ${\mu }_{N}/{\mu }_{N0}$=1.5, $\delta$ =0.25, $\gamma =0.10$, *a* = 0.5 and ${I}_{L}$ =0.04. The study's findings indicate that the existing sampling strategy under traditional statistics is less effective in terms of sample size than the expected sampling plan under indeterminacy. Figure 1 shows the operating characteristic (OC) curve of the WD plan for the conditions when $\gamma =0.10;\delta =0.10, \beta =2.0$ and $a=0.50$. Therefore, in order to test the null hypothesis, the proposed plan needs a lower ASN than the existing plan ${H}_{0}:{\mu }_{N}={\mu }_{N0}$ (Fig. 2). When there is uncertainty, the industrialist can implement the suggested plan faster and with less effort. The ASN performance of MDS design values for $\beta =\{\mathrm{2,1.0}\}$ and $\delta$ =0.05 are displayed in Figs. 3 and 4. In these figures the ratio ${\mu }_{N}/{\mu }_{N0}$ is displayed on horizontal axis and ASN is given on vertical axis. These figures indicate that the existing sampling strategy under traditional statistics is less effective in terms of ASN as compared with the proposed sampling plan under indeterminacy.

The likelihood of MDS acceptance at different levels of indeterminacy is depicted in Fig. 1.

The Fig. 2 shows that probability of acceptance is higher using MDS than the existing sampling plan.

## LED manufacturing process data illustration

Park et al.^46^ and Jin et al.^47^ mentioned that quantum dot light-emitting diodes have uncertainty and inaccurate measurements of device parameters. Let's consider a case study on the production of light emitting light-emitting diodes (LEDs) that focuses on the luminous intensities of LED sources in order to illustrate the use of the provided methodologies. The operational process of light-emitting diode with the help of images can be seen in (https://eepower.com/industry-articles/an-introduction-to-light-emitting-diodes/#). The justification for the process distribution is done and evidence that it resembles the Weibull distribution quite closely. The luminous intensities of LED data has been taken from^48^ and they showed that the process distribution to be fairly close to the Weibull distribution. The steady process is sampled using *n* = 30 sample size. Due to the inevitable degree of imprecision in the data provided by a specific LED's luminous intensity, the luminous intensities of light-emitting diodes are supplied as lower and upper bounds as well as a point estimate, which are as follows:

[2.163, 3.068], [5.972, 8.150], [1.032, 2.642], [0.628, 1.735], [2.995, 5.066], [3.766, 6.212], [0.974, 2.045], [4.352, 5.988], [3.920, 6.121], [1.375, 3.086], [0.618, 2.217], [4.575, 6.734], [1.027, 3.116], [6.279, 9.435], [2.821, 5.272], [7.125, 9.044], [5.443, 7.395], [1.766, 2.638], [7.155, 8.352], [0.830, 2.541], [3.590, 4.899], [5.965, 8.019], [3.177, 4.213], [4.634, 7.058], [7.261, 8.871], [2.247, 4.128], [6.032, 8.529], [4.065, 7.480], [5.434, 7.655], [1.336, 3.284].

The maximum distance between the real-time data and the fitted of WD is found from the Kolmogorov–Smirnov test statistic as [0.12964, 0.14889] and the *p*-value is [0.6473, 0.4742]. It is established that the luminous intensities of light-emitting diodes data drawn from the WD with shape parameter ${\widehat{\beta }}_{N}$= [1.75456, 2.47762] and scale parameter $\widehat{\alpha }=[4.09506, 6.23939]$. As a result, WD is a good fit for data on the luminous intensities of light-emitting diodes. Table 5 lists the plan parameters for this shape parameter. The form parameter for the suggested strategy is ${\widehat{\beta }}_{N}$= [1.75456, 2.47762] when ${I}_{L}$=0.2918. Assume that engineering administrators want to test ${H}_{0}:{\mu }_{N}=[3.6186, 5.4998]$ using the suggested sample scheme when ${I}_{L}$=0.2918,$\gamma =0.10$, ${\upmu }_{{\text{N}}}/{\upmu }_{0{\text{N}}}$=1.5, $a$=0.5 and $\delta$=0.10. From Table 5, it can be noted that ${c}_{1}=6$, ${c}_{2}$=10, *m* = 1 and ASN = 25.

**Table 5:** The MDS design values for $\beta =1.7546$ and ${I}_{L}$=0.2918.

$\delta$	$\frac{{\upmu }_{{\text{N}}}}{{\upmu }_{0{\text{N}}}}$	a = 0.5					a = 1.0				
		${c}_{1}$	${c}_{2}$	$m$	$L\left({p}_{1}\right)$	ASN	${c}_{1}$	${c}_{2}$	$m$	$L\left({p}_{1}\right)$	ASN
0.25	1.5	4	14	2	0.9158	15	2	12	2	0.9150	3
	1.6	3	13	2	0.9284	13	1	11	1	0.9056	2
	1.7	2	4	2	0.9144	9	1	11	1	0.9316	2
	1.8	2	4	2	0.9413	9	1	11	1	0.9500	2
	1.9	1	3	1	0.9121	7	1	11	1	0.9631	2
	2.0	1	3	1	0.9319	7	1	11	1	0.9724	2
0.10	1.5	6	10	1	0.9023	25	4	5	1	0.9075	6
	1.6	5	15	2	0.9121	21	3	13	1	0.9310	5
	1.7	4	14	2	0.9218	18	3	13	1	0.9592	5
	1.8	3	13	2	0.9139	15	2	12	1	0.9255	5
	1.9	2	8	1	0.9066	13	2	12	1	0.9488	4
	2.0	2	12	2	0.9130	12	2	12	1	0.9647	4
0.05	1.5	8	12	1	0.9045	33	6	16	1	0.9443	9
	1.6	6	10	1	0.9165	27	5	15	1	0.9482	8
	1.7	5	15	2	0.9146	23	3	13	2	0.9266	5
	1.8	4	14	2	0.9152	20	2	12	1	0.9255	4
	1.9	3	5	1	0.9178	17	2	12	1	0.9488	4
	2.0	3	5	1	0.9443	17	2	12	1	0.9647	4

The created MDS sampling plan could function as follows: if the average luminous intensities of the light-emitting diodes in 6 measurements are greater than or equal to 23.5255 luminous intensities of the light-emitting diodes, accept the null hypothesis ${H}_{0}:{\mu }_{N}=[3.6186, 5.4998]$. For the batch of light-emitting diodes, a sample of 25 light-emitting diodes with varying luminous intensities will be chosen at random, using null hypothesis ${H}_{0}:{\mu }_{N}=[3.6186, 5.4998]$. The lot of light-emitting diodes will be allowed if the average luminous intensities of the light-emitting diodes prior to $[3.6186, 5.4998]$ are less than or equal to 6 measures, and the lot of light-emitting diodes will be denied if it is larger than 10 measurements. A property of the current batch of light-emitting diodes will be delayed until the testing of the previous lot of light-emitting diodes if the luminous intensities of the light-emitting diodes are between 6 and 10 measurements. The average luminous intensities of light-emitting diodes are more than equal to $[3.6186, 5.4998]$ in more than 17 measurements, which means the assertion that they are ${H}_{0}:{\mu }_{N}=[3.6186, 5.4998]$ might be disproven based on the evidence. Whereas, when compared with the existing MDS plan to test ${H}_{0}:{\mu }_{N}=[3.6186, 5.4998]$ when ${I}_{L}\hspace{0.17em}$= 0,$\gamma =0.10$, ${\upmu }_{{\text{N}}}/{\upmu }_{0{\text{N}}}$=1.5, $a\hspace{0.17em}$= 0.5 and $\delta \hspace{0.17em}$= 0.10 the plan parameters are obtained as ${c}_{1}=7$, ${c}_{2}\hspace{0.17em}$= 16, *m* = 2 and ASN = 53. Which means that the lot of light-emitting diodes will be allowed if the average luminous intensities of the light-emitting diodes prior to $[3.6186, \, 5.4998]$ are less than or equal to 7 measures, and the lot of light-emitting diodes will be denied if it is larger than 16 measurements. A property of the current batch of light-emitting diodes will be delayed until the testing of the previous two lot of light-emitting diodes if the luminous intensities of the light-emitting diodes are between 7 and 16 measurements. The average luminous intensities of light-emitting diodes are more than equal to $[3.6186, \, 5.4998]$ in more than 17 measurements, which means the assertion that they are ${H}_{0}:{\mu }_{N}=[3.6186, \, 5.4998]$ might be rejected. Thus the proposed MDS sampling is performing well as compared with existing MDS sampling plan with respect to ASN. Therefore, engineer administrators could notify the government that light-emitting diode average luminous intensities have reached an unacceptable level. The proposed sample plan is useful in engineering applications, specifically luminous intensities of light-emitting diodes, to determine average luminous intensities of diodes, which is important for any government to do when making policy judgments.

## Concluding remarks

A detailed analysis of the luminous intensities of light-emitting diodes for the Weibull distribution is provided based on an indeterminacy scenario for a time-truncated MDS sampling design. The sampling plans' amounts are set at the previously specified values of the indeterminacy parameter. Comprehensive tables containing the values of the known indeterminacy constants are supplied for the convenience of the researchers. The recently created MDS sampling design based on indeterminacy is compared with the current sampling techniques based on classical statistics. The results show that the created MDS sampling plan under indeterminacy is more rational than the existing SSP under indeterminacy as well as the conventional MDS sampling plans. Furthermore, it is less expensive to run the generated MDS under indeterminacy than the SSP. It's important to keep in mind that the indeterminacy parameter is a major factor in lowering ASN values; hence an increase in the indeterminacy value will unavoidably result in a rise in ASN values. The MDS sample plan created under indeterminacy is therefore more advantageous to scientists, particularly industry practitioners, who are studying or testing sensitive topics that require additional funds and expert researchers. As a result, it is authorized to test light-emitting diode average luminosities using the MDS sampling strategy, which was created in the event of uncertainty. Confirmation is shown by the example employing light-emitting diode data for light intensities for the MDS sampling approach under indeterminacy. Under indeterminacy, other researchers working across distinct domains would follow the standard MDS sampling procedure. The control chart approaches according to multiple dependent state sample plans will be considered the ones in the following study project to monitor the mean. The control chart addresses based on multiple dependent state sample plans would be considered in the subsequent research project to monitor the mean.

## Data Availability

The data is available from the Muhammad Aslam upon the request.
